# Editorial: Association of novel anthropometric indexes with metabolic syndrome

**DOI:** 10.3389/fendo.2022.951571

**Published:** 2022-08-08

**Authors:** Patricia Khashayar, Ozra Tabatabaei-Malazy, Mostafa Qorbani, Roya Kelishadi

**Affiliations:** ^1^ Center for Microsystems Technology, Imec & Ghent University, Zwijnaarde-Gent, Belgium; ^2^ Non-Communicable Diseases Research Center, Endocrinology and Metabolism Population Sciences Institute, Tehran University of Medical Sciences, Tehran, Iran; ^3^ Non-Communicable Diseases Research Center, Alborz University of Medical Sciences, Karaj, Iran; ^4^ Department of Pediatrics, Child Growth and Development Research Center, Research Institute for Primordial Prevention of Non-Communicable Disease, Isfahan University of Medical Sciences, Isfahan, Iran

**Keywords:** obesity, anthropometry, waist-to-height ratio, waist circumference, body mass index, adiposity, metabolic syndrome, cardiometabolic risk factors

The prevalence of being overweight and obese has been increasing in people living in both developed and developing countries (1). Obesity is linked to cardiometabolic risk factors, including insulin resistance, type 2 diabetes, hyperlipidemia, and hypertension, all of which can lead to cardiometabolic diseases (2, 3). Body Mass Index (BMI), defined as weight (kg)/height (m)2 has been used since 1970 to classify obesity in adults. One of the main limitations of BMI is the fact that it measures excess weight rather than excess fat (4). Recently, novel anthropometric measures, such as body shape index, hip index, body surface area, vertical trunk circumference, and visceral adiposity index, have been developed to overcome the BMI limitations (5). Due to the strong association between obesity and a long list of complications, which all are responsible for the increased risk of morbidity and mortality, the current Research Topic entitled “Association of Novel Anthropometric Indexes with Metabolic Syndrome” was initiated. Two main objectives of the current Research Topic are studying the link between novel anthropometric indices and cardiometabolic risk factors, and comparing the association between novel and traditional anthropometric indices and cardiometabolic risk factors.

Overall, the present Research topic collection is compiled from authors from various countries and examines the relationship between obesity and cardiometabolic risk factors such as insulin resistance, and type 2 diabetes.

In the first article of this Research Topic, by Xi et al., an association was found between being overweight and a 94% increased risk of hypertension in 30,617 twin individuals selected from the Chinese National Twin Registry (CNTR). They comment that common genetic predisposition and early-life environments are linked with obesity and hypertension; the effect of the environment however proved to be less significant. In a study by Kim et al. non-diabetic subjects scheduled to undergo bariatric surgery, showed that increased adiposity, visceral fat area (VFA), and the homeostatic model for insulin resistance (HOMA-IR) were independent risk factors of polyneuropathy (PN). As for the participants with diabetes, however, the role of fibrosis detected as increased expression of fibrotic genes such as TIMP1 was more significant. These studies, along with many others, highlight the need for a more accurate tool to diagnose the high-risk obese individuals who may benefit from intervention. Despite the common usage of BMI in determining obesity, many studies have pointed out its shortcomings, such as incapability in distinguishing adipose mass from muscle and thus differentiating between low- and high-risk phenotypes of obesity. As a result, several articles in this Research Topic (Liu et al., Khonsari et al., Wang et al., Sun et al., Guo et al., Li et al.) investigated the association between novel anthropometric indexes and cardiometabolic risk factors for screening and the management of obesity.

In this regard, some articles have used a combination of existing markers with BMI to improve its efficacy. In the Liu et al. study, BMI is combined with waist circumference to assess 35,557 adults (51.1% women with a total mean age of 44.9 years) from the National Health and Nutrition Examination Survey (NHANES 1999-2014). The authors confirm waist-BMI ratio to be a promising marker for determining the high-risk phenotype of obesity. They conclude this ratio to be a better discriminatory proxy of mortality (all cause and cardiovascular mortality) compared with each of the markers alone.

Another novel anthropometric index is normal weight obesity (NWO), which shows normal BMI but with high-fat percentage. Khonsari et al. in their systematic review/meta-analysis study, pooled the results of the studies assessing the correlation between NWO and cardiometabolic risk factors. In their meta-analysis, a significant association is observed between NWO and cardiometabolic risk factors. They assert body fat percentage to be a better index than BMI for obesity risk assessment.

Liu et al. reported an increased risk of diabetes in hypertensive individuals with high adiposity index, defined by high waist-to-height ratio (WHtR). They suggest WHtR as a non-invasive, cost-saving public health tool to assess diabetes risk among the hypertensive adult population. As pediatric obesity is also associated with increased incidence of cardiometabolic risk factors, the usefulness of WHtR is also assessed in children and adolescents. In a cross-sectional population-based study, WHtR is used to identify the presence of left ventricular hypertrophy (LVH) and geometric (LVG) as markers of cardiac structural damage (Wang et al.). They report WHtR to be a similar or better predictive tool compared with BMI and both to be stronger than waist circumference in identifying children at risk of subclinical cardiac structural damage in adulthood.

Others, however, have focused on newer markers to assess body fat and replace BMI. In a systematic review, Sun et al. studied the value of tri-ponderal mass index, calculated as weight (kg)/height (m3) and defined it as a new indicator for adiposity and obesity-related cardiovascular risk factors (CVRFs) in children and adolescents. The results of this systematic review revealed that tri-ponderal mass index had a similar or better ability to predict body fat compared with BMI. Despite being similar to BMI in identifying MetS, tri-ponderal mass index is suggested to be a useful tool when used in combination with other indicators (e.g., BMI and waist circumference). In addition, limited evidence shows that tri-ponderal mass index does not perform better than BMI in identifying specific CVRFs, including insulin resistance, high blood pressure, dyslipidemia, and inflammation in children and adolescents, as well as CVRFs in adults.

Visceral adipose (VA) tissue defined as an “ectopic fat” can increase the risk of metabolic syndrome (MetS) by stimulating systemic inflammation, insulin resistance, and metabolic profiles. From among the VA tissue deposits, perirenal fat thickness can be easily measured using ultrasound, CT, and MRI scanning. In addition to its special anatomical structure, perirenal fat thickness can modulate the metabolism system. It can, thus, be a promising surrogate marker in identifying MetS. Guo et al. reported a significant association between perirenal fat thickness and MetS, as well as its components in individuals with newly diagnosed diabetes.

In the ninth article, Li et al. assessed the association between four anthropometric indexes including lipid accumulation products, waist-triglyceride index, visceral obesity index, triglyceride and glucose index, and MetS in the National Health and Nutrition Examination Survey (NHANES). The NHANES, a population-based study conducted between 1996-2006, is representative of the American adults. The authors concluded that the products of lipid accumulation were the strongest predictor of MetS in both sexes, suggesting them to be a more suitable tool to predict MetS in the clinical setting.

In another attempt, researchers looked into circulating biomarkers. In the article by Hu et al., increased serum levels of C1q/TNF-related protein 7 (CTRP7) were reported in MetS patients, suggesting they are a possible biomarker for detecting metabolic diseases. This is in line with previous animal studies that had linked CTRP7 with energy metabolism. In this study, serum levels of CTRP7 were confirmed to be significantly higher in MetS patients, showing a positive correlation with waist circumference, blood pressure, fasting blood glucose, 2h-blood glucose and triglyceride, but a negative correlation with HDL-C and adiponectin. They also confirmed a strong link between CTRP7 and metabolism-related genes and signal pathways by performing interventional studies (HEC, OGTT and lipid infusion) in healthy individuals.

In the eleventh article, an abnormal amino acid profile is reported in a cohort on the Chinese Han population (Sun et al.). This finding is the continuation of studies linking certain plasma amino acids with visceral obesity, insulin resistance, future development of diabetes, and cardiovascular diseases. Targeted liquid chromatography/tandem mass spectrometry (LC/MS) combined with principal component analysis (PCA) suggests a profile consisting of 12 amino acids (isoleucine, leucine, valine, tyrosine, tryptophan, glutamic acid, aspartic acid, alanine, histidine, methionine, asparagine, and proline) as capable of assessing and monitoring of MetS risk. Reduced taurine levels show promising results for early diagnosis of the disease.

Galectin-3-binding protein (GAL-3BP) is a glycoprotein known for its functions in innate immunity and is a potential mediator of adipose inflammation in obesity. The study by Zhen et al. showed a positive association between serum GAL-3BP and MetS in the Chinese population. This association is more significant among postmenopausal women.

Childhood obesity, regardless of age, results in adulthood obesity. One of the factors associated with early childhood obesity is maternal smoking. Hirai et al. reported urinary cotinine concentration as an accurate and quantitative marker for maternal smoking and childhood obesity among 89,617 mother-infant singleton pairs. They used the concentrations of cotinine in the mothers’ urine, rather than their smoking classes, to predict childhood obesity in a dose-dependent manner.

Infrared thermography (IRT) is a non-contact and non-invasive technique, in which infrared emanated radiation from the body is captured and converted into temperature. This is then used for early screening of numerous diseases such as breast cancer and diabetic neuropathy. The skin temperature affected by local blood perfusion can, to some extent, determine tissue activity. For example, skin temperature of the anterior supraclavicular can reflect metabolic changes by detecting activated brown adipose tissue (BAT). Gao et al. assess the association of IRT and temperature distribution of the face, palms, feet, and the trunk area with MetS. They report a positive correlation between the temperature of face, palms, and dorsum of feet and the number of MetS components. As for the temperature of the anterior trunk, they show a negative association.

To conclude, all articles in this Research Topic collection propose novel anthropometric indexes as well as their association with MetS. This Research Topic has a great scientific impact and points out the importance of early detection of obesity in all ages and both genders. Moreover, they highlight current research gaps, paving the way for future research on the topic as well as practical strategies to overcome the existing shortcomings.

## Author contributions

All authors listed have made a substantial, direct, and intellectual contribution to the work and approved it for publication.

## Conflict of interest

The authors declare that the research was conducted in the absence of any commercial or financial relationships that could be construed as a potential conflict of interest.

## Publisher’s note

All claims expressed in this article are solely those of the authors and do not necessarily represent those of their affiliated organizations, or those of the publisher, the editors and the reviewers. Any product that may be evaluated in this article, or claim that may be made by its manufacturer, is not guaranteed or endorsed by the publisher.
